# Circular of the Permanent Secretary

**Published:** 1878-05

**Authors:** 


### American Medical Association.

We publish the following which speaks for itself:

PHILADELPHIA, 1400 Pine Street,<br>S. W. corner Broad.

The Twenty-ninth Annual Session will be held in the city of Buffalo, N. Y., on Tuesday, Wednesday, Thursday, and Friday, June 4, 5, 6 and 7, 1878, commencing on Tuesday at 11 A. M.

"The delegates shall receive their appointment from permanently organized State Medical Societies, and such County and District Medical Societies as are recognized by *representation in their respective State Societies*, and from the Medical Department of the Army and Navy of the United States."

"Each State, County, and District Medical Society entitled to representation shall have the privilege of sending to the Association one delegate for every ten of its regular resident members, and one for every additional fraction of more than half that number: *Provided*, however, that the number of delegates for any particular State, territory, county, city, or town shall not exceed the ratio of one in ten of the resident physicians who may have signed the Code of Ethics of the Assotion,"

☞ Secretaries of Medical Societies as above designated are earnestly requested to forward, *at once*, lists of their delegates.

Will you kindly send to the undersigned a list of your members with their residences, in order that a correct record may be made of all who are in affiliation with this body?

SECTIONS.

"The Chairmen of the several sections shall prepare and read in the general sessions of the Association, papers on the advances and discoveries of the past year in the branches of science included in their respective Sections. * * *" —By-Laws, Art. II., Sect. 4.

Practice of Medicine, Materia Medica, and Physiology:
Dr. A. L. Loomis, New York, *Chairman*.
Dr, J. H. Etheridge, Chicago, Ill., *Secretary*.

Committee appointed to report to this Section:

On Clinical and Meteorological Records:
Dr. N. S. Davis, Illinois, *Chairman*.

Obstetrics and Diseases of Women and Children:
Dr. E. W. Jenks, Detroit, Mich., *Chairman*.
Dr. H. O. Marcy, Cambridge, Mass., *Secretary*.

Surgery and Anatomy:
Dr. Henry H. Smith, Philadelphia, Pa., *Chairman*.
Dr. E. T. Easley, Little Rock, Ark., *Secretary*,

Medical Jurisprudence, Chemistry, and Psychology:
Dr. Walter Kempster, Oshkosh, Wis., *Chairman*.
Dr. E. A. Hildreth, Wheeling, W. Va., *Secretary*,

State Medicine and Public Hygiene:
Dr. J. L. Cabell, University of Va., *Chairman*.
Dr. E. J. Marsh, Paterson, N. J., *Secretary*.

The following Committees are expected to report:—

On Prize Essays:
Dr. E. M. Moore, Rochester, N. Y,, *Chairman*.

On Necrology:
Dr. J. M. Toner, Washington, D. C., *Chairman*.

On Catalogue and National Library:
Dr. H. C. Wood, Pa., *Chairman*.

On Recommendations in President Bowditch's Address:
Dr. N. S. Davis, Illinois, *Chairman*.

To be acted upon:—

*Changes in By-Laws*, proposed by Committee on Nominations in 1877.

"Under Art. II., Sections, 10th line from top, the word *Essayist* shall be introduced immediately after the word 'Chairman,' so as to read as follows: 'The Chairman, Essayist, and Secretary of the several sections shall, like other officers, of the Association, be nominated," etc.

Same Article, line 22d *et seq.*, to read as follows: "The Chairmen of the several Sections shall preside at the meetings of their respective Sections. The Essayists shall prepare and read in the general sessions of the Association papers on some subject to be selected by themselves, but relating to one or more of the branches of science included in their respective Sections; the reading of such papers not to occupy longer than forty minutes for each."

Amendment proposed by Dr. S. C. Busey:—

*Plan for Organization of the American Medical Association.*

The general meetings of the Association shall be restricted to the morning session; and the afternoon sessions, commencing at three o'clock, shall be devoted to the hearing of reports and papers, and their consideration, in the following Sections:—

1. Practical Medicine, Materia Medica, and Physiology.
2. Obstetrics and Diseases of Women and Children.
3. Surgery and Anatomy.
4. Medical Jurisprudence, Chemistry, and Psychology.
5. State Medicine and Public Hygiene.

The Sections shall be organized by selecting from the permanent membership such members as may have acquired distinction in the branches of medical science assigned to the respective Sections, such selections to be made from the roster of permanent membership next preceding the adoption of this amendment, by a committee consisting of the chairmen and so many of the ex-chairmen of the respective Sections for the three years next preceding as may be present at the time of the adoption of this amendment; provided, however, that no one shall be thus made a member of more than one Section.

Only permanent members shall be eligible to membership of the Sections; and annually, as hereinafter provided, each Section may elect such as may be qualified by three years' consecutive membership, and who may have acquired distinction in the branches assigned to the Section. Forfeiture of permanent membership shall forfeit membership of Sections, but such forfeiture shall not prevent a re-election.

The officers of the Section shall consist of a Chairman, Secretary, a Committee on Business, a Committee on Membership, and a Committee on Essays. The Chairman and Secretary shall be elected by a majority of the ballots cast by the members of the Section, and shall hold their offices until the close of the business of the annual meeting next succeeding their election. The committees shall be appointed by the Chairman, and hold office for one year or until their successors are appointed.

The Chairman shall preside at all the meetings of the Section, perform all the duties ordinarily pertaining to the duties of a presiding officer; shall exercise general supervision over the business of the Section; see that the duties of the officers and committees are properly discharged, and shall prepare and read, in general sessions of the Association, a paper on the advances and discoveries of the past year in the branches included in the Section; the reading of such paper shall not occupy more than forty minutes.

It shall be the duty of the Secretary to keep a correct record of the proceedings of the Section and report the same to the Permanent Secretary; to revise and correct the reports of the stenographer, and deliver the same to the Committee on Essays; and to perform such other duties as may properly appertain to the office of Secretary.

The Committee on Business of each Section shall consist of three members, to be selected, when practicable, from the members residing in or near the city in which the meeting of the Association is to be held.

It shall be the duty of this Committee to prepare business for the Section, and, with that in view, to select two or more subjects appropriate to the Section, and also to appoint from the members of the Section as many members, to each of whom a subject shall be assigned for report thereon, which report shall be prepared and submitted to the Section at the meeting next ensuing. Voluntary papers may, with the approbation of the Committee, be submitted by any member of, or delegate to, the Association. All papers and reports must be sent to the Committee at least one month before the meeting, and it shall be the duty of the Committee, after careful examination, to fix the time and order of presentation, and to prepare a memorandum of the titles, together with the main points set forth in the argument, which shall be printed and distributed to the members of the Sections by the Assistant Secretary.

The Committee on Membership of each Section shall consist of five members. It shall be the duty of this Committee annually to examine the roster of permanent membership, and recommend for election to membership of the respective Sections such as may be eligible and deemed qualified. It shall also examine the roster of membership of the Section, make all necessary corrections of names and addresses, erase from the list the names of all who may have forfeited their membership, and designate the deceased members.

The Committee on Essays shall be composed of three members. To this Committee shall be referred for examination all papers read before the Section, and all debates. The Committee will be allowed thirty days, at the expiration of which time it shall forward the papers to the Committee on Publication, with such recommendation as may be deemed proper; but no paper or discussion, either in whole or in part, shall be published in the Transactions without the recommendation in writing of said Committee on Essays.

The Committee of Arrangements shall provide for each Section a competent stenographer, who shall furnish the Secretary with a full verbatim report of the debates of the Section.

No member shall address the Section more than once upon the same subject, nor speak longer than fifteen minutes without unanimous consent.

The Permanent Secretary shall prepare annually a list of the members of each Section, which shall be published in the Transactions, and shall furnish each delegate with a printed plan of organization and by-laws, together with rosters of the permanent and Section membership.

Amendment proposed by Dr. T. D. Fitch :—

" Strike out from By-laws the whole of fifth paragraph, Sect. II. ' Papers appropriate to the several Sections, in order to secure consideration and action, must be sent to the Secretary of the appropriate Section at least one month before the meeting which is to act upon them. It shall be the duty of the Secretary to whom such papers are sent, to examine them with care, and, with the advice of the Chairman of his Section, to determine the time and order of their presentation, and give due notice of the same; and, after their full examination and discussion by the Section, they shall be sent to the Permanent Secretary of the Association.' "

" Strike out all of third paragraph, Section VIII, " It shall be the dnty of every member of this Association, who learns that any existing medical school departs from the published conditions of graduation, to report the fact at the annual meetings; and, on proof of the fact, such school shall be deprived of its representation in this body."

Strike out all of second paragraph, Section IX, " This Association recognizes as a ' regular organized' medical college one that has been represented at any meeting, and that complies with the rules and directions found in the published *Transactions*, vol. xiii. page 33."

Amendment offered by Dr. X. C. Scott, and others :—

" Add to the five existing Sections a Section for Opthalmology, Otology, and Laryngology, which shall be known and designated as Section 6."

---

It is probable that several railroads will carry delegates to Buffalo and return for one and one-third fare. Such roads as agree so to do will be announced in the Journals.

WM. B. ATKINSON, M. D.,
*Permanent Secretnry.*

